# Whole genome sequencing of *Oryza sativa* L. cv. Seeragasamba identifies a new fragrance allele in rice

**DOI:** 10.1371/journal.pone.0188920

**Published:** 2017-11-30

**Authors:** Ganigara Bindusree, Purushothaman Natarajan, Sukesh Kalva, Parani Madasamy

**Affiliations:** Genomics Laboratory, Department of Genetic Engineering, SRM University, Kattankulathur, India; National Institute for Plant Genome Research, INDIA

## Abstract

Fragrance of rice is an important trait that confers a large economic benefit to the farmers who cultivate aromatic rice varieties. Several aromatic rice varieties have limited geographic distribution, and are endowed with variety-specific unique fragrances. *BADH2* was identified as a fragrance gene in 2005, and it is essential to identify the fragrance alleles from diverse geographical locations and genetic backgrounds. Seeragasamba is a short-grain aromatic rice variety of the indica type, which is cultivated in a limited area in India. Whole genome sequencing of this variety identified a new *badh2* allele (*badh2-p*) with an 8 bp insertion in the promoter region of the *BADH2* gene. When the whole genome sequences of 76 aromatic varieties in the 3000 rice genome project were analyzed, the *badh2-p* allele was present in 13 varieties (approximately 17%) of both indica and japonica types. In addition, the *badh2-p* allele was present in 17 varieties that already had the loss-of-function allele, *badh2-E7*. Taken together, the frequency of *badh2-p* allele (approximately 40%) was found to be greater than that of the *badh2-E7* allele (approximately 34%) among the aromatic rice varieties. Therefore, it is suggested to include *badh2-p* as a predominant allele when screening for fragrance alleles in aromatic rice varieties.

## Introduction

Rice is a major staple food feeding hundreds of millions of people in Asia [1]. The unique fragrance of aromatic varieties makes rice appealing to people in other parts of the world who do not consume it as a staple food. Even in traditional rice-eating countries, highly-priced rice dishes are prepared using aromatic rice varieties. Therefore, the fragrance of aromatic rice varieties is an economically important trait that fetches premium price in domestic as well as international markets. In fact, many rice-growing countries earn huge foreign exchange by exporting aromatic rice varieties to developed countries [2]. Several long-grain and short-grain aromatic rice varieties with considerable variations in fragrance are available in the market catering to consumer preferences. Some of the aromatic rice varieties are traditional cultivars grown in very limited areas, and are known for their unique fragrance [3, 4, 5].

Beyond its importance as the world’s premier food crop, rice is also an excellent model plant for crop genomics [6]. Earlier works on rice genomics analyzed genome-wide genetic variations to understand gene functions related to agronomic traits. Genetic studies have shown that the fragrance of aromatic rice is controlled by the *fgr* recessive gene [7, 8], which was later identified to be a gene coding for betaine aldehyde dehydrogenase 2, *BADH2* [9]. The first loss-of-function allele in *BADH2* (*badh2*.*1* or *badh2-E7*) was identified as an 8-bp deletion in the seventh exon [9]. Subsequently, eighteen *badh2* alleles associated with the rice fragrance were reported [10, 11]. While a majority was loss-of-function mutations due to InDels or SNPs in coding regions, non-synonymous mutations in coding regions, mutations in intron-exon junction, promoter, and 5’UTR were also reported. Such variations may account for the spectrum of unique fragrances observed in aromatic rice varieties. Therefore, it is important to characterize the *badh2* alleles from diverse aromatic rice varieties to generate a panel of fragrance alleles for breeders to choose the desired one.

Seeragasamba (also called Jeeraga Samba) is a short-grain aromatic rice variety that is cultivated in select regions of Tamil Nadu, India [12]. Sakthivel et al. [13] screened the *badh2-E7* allele in 47 aromatic rice varieties from India including Seeragasamba. Though *badh2-E7* allele was present 42 varieties, it is not clear if Seeragasamba had this allele or not. Another study reported that Seeragasamba did not have the *badh2-E2*, *badh2-E7* or *badh2-E8* allele for which screening was undertaken [14]. Therefore, we analyzed the entire *BADH2* gene and its promoter from the whole genome sequence of Seeragasamba; a new *badh2* allele was identified. Evidence for this new allele in other aromatic rice varieties was obtained based on the whole genome sequences of 76 aromatic rice varieties from the 3000 rice genome project.

## Materials and methods

### Whole-genome sequencing of *Oryza sativa* L. cv. Seeragasamba

Seeds of Seeragasamba were obtained from a farmer’s field in Tamil Nadu, India. Seedlings were grown in pots under greenhouse conditions. Genomic DNA was isolated from young leaves using the cetyl trimethyl ammonium bromide (CTAB) method [15]. Quantity and quality of the genomic DNA were assessed using spectrophotometer, fluorimeter, and agarose gel electrophoresis. Preparation of paired-end library and sequencing were carried according to the manufacturer’s protocol (Illumina Inc., USA). Paired-end reads were extracted in FASTQ format for further downstream analysis.

### Genome mapping and variant calling in Seeragasamba

Quality of the raw paired-end reads was assessed using the FastQC tool [16]. Adapter sequences were trimmed off using the cutadapt tool [17]. The paired-end reads were further filtered by retaining the bases with a minimum Phred quality score of 30 using sickle master (https://github.com/najoshi/sickle). The quality filtered reads were mapped to the latest unified build release Os-Nipponbare-Reference-IRGSP-1.0 [18] using Burrows-Wheeler alignment (BWA) software [19]. The aligned reads in the SAM file were sorted using the SortSam function of the Picard tool v1.118 (https://broadinstitute.github.io/picard/). The sorted SAM file was converted to BAM file using SAMtools v0.1.19 [20] for variant calling. Variant calling was carried out using mpileup application of SAMtools [20] setting at default parameters. The variants were further filtered based on the following criteria: (1) base quality ≥ 30, (2) number of reads per base between 5 and 75, (3) variant quality ≥ 90, (4) mapping quality ≥ 60, and (5) distance of adjacent variant ≥ 5. The filtered variants were extracted in Variant Call File (VCF) format and annotated using the SnpEff V3.6 tool [21] and rice7 gene model database (http://sourceforge.net/projects/snpeff/files/databases/v3_6/snpEff_v3_6_rice7.zip). The total numbers of variants were segregated as SNPs and InDels. Variants in the genes, and other genomic regions were annotated as genic and intergenic variants, respectively. According to the location of the genic SNPs, they were further classified as CDs, UTRs (5'UTRs and 3'UTRs), introns and regulatory sequences. The SNPs found in the coding regions were categorized as non-synonymous (causes change in amino acid), synonymous (causes no change in amino acid), stop loss (removes the existing stop codon), stop gain (introduces a stop codon), start gain (introduces a start codon), and start loss (removes an existing start codon). The SNPs were differentiated as transition (C/T and G/A) and transversion (C/G, T/A, A/C and G/T) SNPs.

### Analysis of variants in the aromatic rice varieties of 3000 rice genome project

Whole genome data for 3024 rice varieties were made available from the 3000 rice genome project [22], which included 76 aromatic rice varieties. A searchable Rice SNP-Seek Database containing about 20 million SNPs obtained after aligning these genomes with the Nipponbare reference was created [23] and further updated to include InDels [24]. Using this database, we have analyzed the variants present in the *BADH2* gene (including -1326 bp upstream region) in all the 76 aromatic rice varieties in the database. VCF containing the SNPs from 39 aromatic rice varieties, which contained either *badh2-E7* or *badh2-p* (identified from the current study) or both alleles were extracted from the Rice SNP-Seek Database, and merged with the SNPs from Seeragasamba. The merged VCF was converted into Genomic Data Structure (gds) format using gdsfmt package of R software (https://www.r-project.org/). Bi-allelic SNPs were extracted from the gds file. SNPRelate program from R package was used to generate dendrogram using identity-by-state (IBS) distance matrix.

## Results and discussion

**Fig 1:**
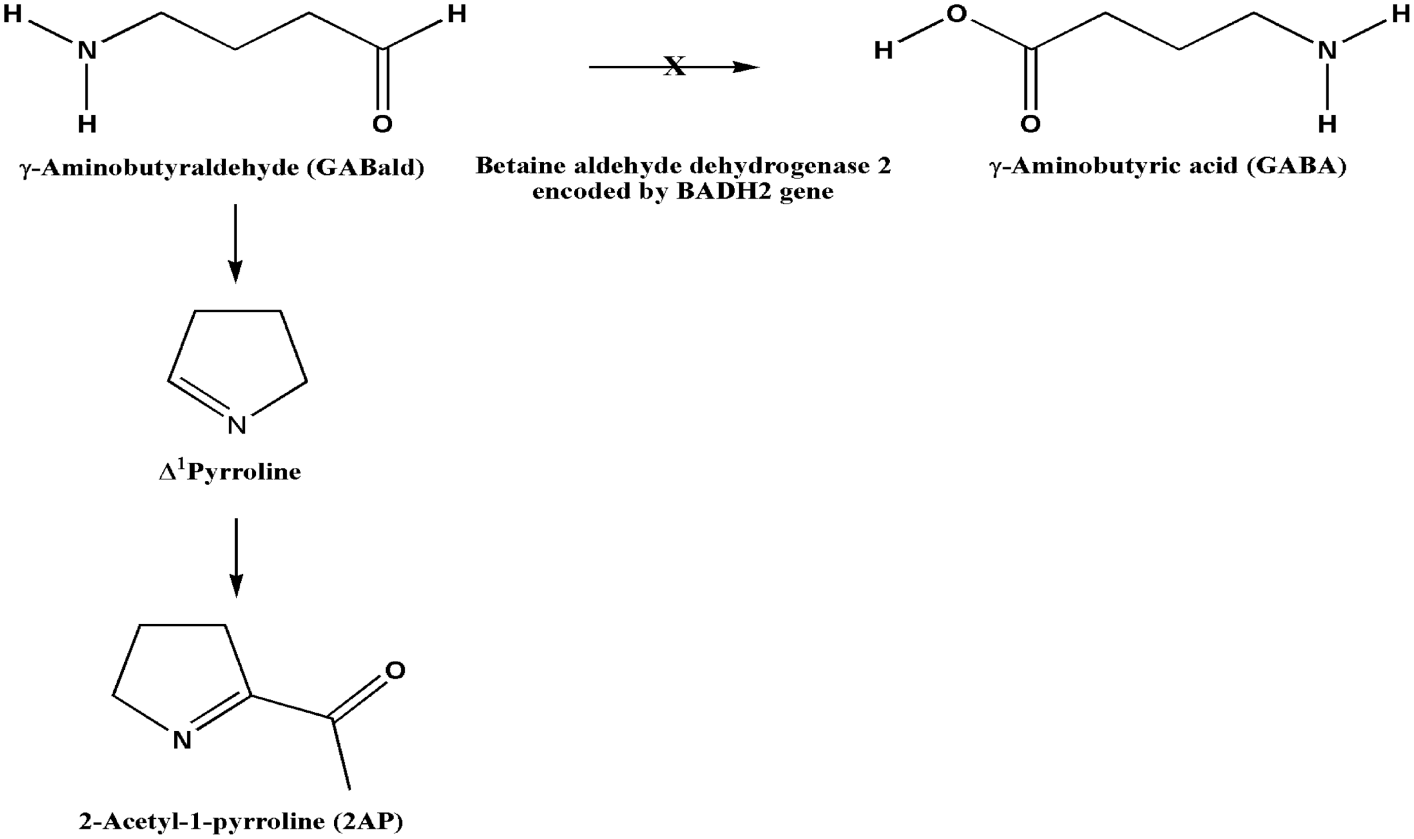
Biosynthetic pathway of 2-acetyl-1-pyrroline (2AP) in rice. Functional *BADH2* converts γ-aminobutyraldehyde (GABald) to γ-aminobutyric acid (GABA). When *BADH2* is partly or fully non-functional, GABald is diverted to the production of Δ^1^pyrroline and 2AP (responsible for fragrance).

The major compound responsible for the fragrance of aromatic rice is 2-acetyl-1-pyrroline (2AP), which is present in all aerial parts of the plant [25, 26]. Normally, the product of *BADH2* gene, betaine aldehyde dehydrogenase 2, converts γ-aminobutyraldehyde (GABald) to γ-aminobutyric acid [27]. GABald is diverted to the production and accumulation of 2AP when *BADH2* is partly or fully non-functional. This gives aromatic rice varieties their characteristic fragrance (Fig 1). *BADH2* in chromosome 8 is a homologue of *BADH1* in chromosome 4, and its size is 6154 bp with 15 exons and 14 introns.

We performed whole genome sequencing of the Seeragasamba rice variety via Illumina sequencing by synthesis method to study the mutations in *BADH2* gene and its promoter. Data from this study was submitted to NCBI under BioProject ID PRJNA324355 and BioSample ID SAMN05200854. Raw reads of this project were deposited in compressed FASTAQ format at SRA database of NCBI with the accession number SRP076132 (http://www.ncbi.nlm.nih.gov/sra/SRP076132).

Whole genome sequencing of the genomic DNA from Seeragasamba produced 42.6 x 10^6^ raw reads with an average read length of 101 bp. The raw reads were quality filtered, and the resulting 38.6 x 10^6^ high-quality reads totaling 4.2 x 10^9^ bp were used for mapping to Nipponbare reference genome. About 30.8 x 10^6^ high-quality reads (79.8%) were uniquely mapped, which covered 86.5% of the reference genome. Chromosome-wide coverage varied between 82.8 and 91.6% in chromosomes 1 and 12, respectively. The initial variant identification yielded 3,166,688 SNPs and 265,109 InDels. Quality filtering of these variants using the five parameters as described in the materials and methods yielded 671,708 and 60,705 SNPs and InDels, respectively. All quality-filtered variants were annotated; detailed classification of the annotated variants is given in Table 1. Detailed analysis of the annotated variants in the genomic region spanning 20,378,646 bp to 20,385,975 bp in chromosome 8 was carried out to identify *badh2* allele responsible for the fragrance of Seeragasamba.

**Table 1:** Annotation of SNPs and InDels in Seeragasamba.

Location/Type of the variant	SNPs	InDels
Intergenic	445,900	8,259
Genic	225,808	52,446
Intron and regulatory sequences	138,005	34,653
UTRs	27,195	11,184
Coding regions (CDs)	60,608	6,609
Synonymous SNPs	25,668	-
Non-Synonymous SNPS	34,940	-
Stop loss	243	-
Stop gain	908	-
Start gain	1055	-
Start loss	95	-
Transition SNPs (Ts)	457,091	-
Transversion SNPs (Tv)	214,617	-
Ts/Tv ratio	2.1298	-

An 8 bp deletion in exon 7 (*badh2*.*1* or *badh2-E7* allele) resulting in a shift of the reading frame and premature termination of translation is the most predominant loss-of-function mutation in *BADH2* gene [9]. However, InDels in exon 1, 2, 4, 5, 8, 12, 13, and 14 were also reported [10, 11, 28, 29, 30, 31]. Two SNPs resulting in non-sense mutations and premature termination of translation were reported in the exon 10 [10, 13]. A mutation in the splice donor site at exon 1–intron 1 was reported in six Japanese aromatic landraces [32]. Reports on mutations in the non-coding regions of *BADH2* are limited. Nankai 138, a Japanese aromatic rice variety, did not have any mutations in the coding region. It was reported to have an 8 bp insertion in the promoter region upstream of the start codon between -1314 and -1315 position and a 3-bp deletion in the 5'UTR from -81 to -83 positions in *BADH2* (*badh2-p-5'UTR* allele) [33].

Seeragasamba also contained the same 8-bp insertion in the promoter region without any mutation in the coding region. It did not have the 3-bp deletion in the 5'UTR. This allele was named as *badh2-p*. Whole genome sequences of 76 aromatic varieties from Bangladesh, Bhutan, India, Iran, Japan, Liberia, Madagascar, Myanmar, Nepal, Pakistan, Philippines, Taiwan, and Thailand were available in the 3000 rice genome project [22]. A detailed variant analysis of *BADH2* gene in these aromatic varieties was carried out, and the presence of *badh2* alleles was documented (Table 2). Twenty-six varieties (approximately 34%) had the *badh2-E7* allele, which was reported to be the most predominant fragrance allele in aromatic rice varieties. No other reported fragrance alleles with mutations in the coding region or non-coding region were found among the 76 aromatic varieties.

**Table 2:** List of *badh2* alleles identified from the whole genome sequences of the aromatic rice varieties in the 3000 rice genome project.

S.No	Name of the variety	Variety Group	Origin	Allele 1	Allele 2
1	P 335::IRGC 69001–1	Aus/boro	Liberia	*badh2-E7*^#^	NIL
2	BASMATI 385	Intermediate type	Pakistan	*badh2-E7*	NIL
3	MADHUWA KARIA::IRGC 16138–2	Basmati/sadri	Nepal	*badh2-E7*	NIL
4	BARA PASHAWARI 390::IRGC 27779–1	Basmati/sadri	Pakistan	*badh2-E7*	NIL
5	BADSHABHOG 4–60::IRGC 37793–2	Basmati/sadri	Bangladesh	*badh2-E7*	NIL
6	MILAGROSA, ZAWA BANDAY	Indica	Philippines	*badh2-E7*	NIL
7	DOMSIAH	Japonica	Iran	*badh2-E7*	NIL
8	POHHERLIMASION::IRGC 62025–1	Basmati/sadri	Nepal	*badh2-E7*	NIL
9	TAROM MOLAII	Intermediate type	Iran	*badh2-E7*	NIL
10	JC 157	Aus/boro	India	*badh2-E7*	badh2-p*
11	JC 1	Basmati/sadri	India	*badh2-E7*	*badh2-p*
12	BASMATI 1::IRGC 27798–1	Basmati/sadri	Pakistan	*badh2-E7*	*badh2-p*
13	HIRA NAKHI::IRGC 70840–1	Indica	India	*badh2-E7*	*badh2-p*
14	KASHA::IRGC 83865–1	Basmati/sadri	Bangladesh	*badh2-E7*	*badh2-p*
15	BASMATI SURKH 161::IRGC 27797–1	Basmati/sadri	Pakistan	*badh2-E7*	*badh2-p*
16	BEGUNBICHI 33::IRGC 29260–1	Basmati/sadri	Bangladesh	*badh2-E7*	*badh2-p*
17	PANKAIT 31::IRGC 29377–1	Basmati/sadri	Bangladesh	*badh2-E7*	*badh2-p*
18	BAJAL::IRGC 45024–2	Basmati/sadri	India	*badh2-E7*	*badh2-p*
19	KEYA NUNIA::IRGC 46117–2	Basmati/sadri	India	*badh2-E7*	*badh2-p*
20	SADRI RICE 1	Basmati/sadri	Iran	*badh2-E7*	*badh2-p*
21	RATO BASMATI::IRGC 59205–1	Indica	Nepal	*badh2-E7*	*badh2-p*
22	CR 44–1::IRGC 45397–1	Basmati/sadri	India	*badh2-E7*	*badh2-p*
23	IET 14720::IRTP 23179-G1	Tropical japonica	Na	*badh2-E7*	*badh2-p*
24	NS 1576::IRGC 68951–1	Indica	Madagascar	*badh2-E7*	*badh2-p*
25	KARNAL LOCAL	Basmati/sadri	India	*badh2-E7*	*badh2-p*
26	SETO JHINUWA::IRGC 62043–1	Indica	Nepal	*badh2-E7*	*badh2-p*
27	CODE NO 31225::IRGC 46865–2	Intermediate type	India	*badh2-p*	NIL
28	ARC 10497	Basmati/sadri	India	*badh2-p*	NIL
29	IRAT 118::IRGC 55785–1	Temperate japonica	Madagascar	*badh2-p*	NIL
30	HALIDA::IRGC 59005–1	Indica	Nepal	*badh2-p*	NIL
31	TAIPEI 167::IRGC 65371–1	Indica	Taiwan	*badh2-p*	NIL
32	HANSRAJ::IRGC 70836–1	Basmati/sadri	India	*badh2-p*	NIL
33	TK RED 35–799::IRGC 38535–1	Basmati/sadri	Bangladesh	*badh2-p*	NIL
34	GUDURA::IRGC 61955–1	Basmati/sadri	Nepal	*badh2-p*	NIL
35	ARC 13502::IRGC 41126–2	Basmati/sadri	India	*badh2-p*	NIL
36	JALADHI 1::IRGC 45858–2	Intermediate type	India	*badh2-p*	NIL
37	T 3::IRGC 46760–2	Basmati/sadri	India	*badh2-p*	NIL
38	ARC 14398::IRGC 41544–2	Basmati/sadri	India	*badh2-p*	NIL
39	ARC 7296::IRGC 20585–1	Basmati/sadri	India	*badh2-p*	NIL

^#^ Bradbury et al. 2005 *badh2-E7* allele

*new allele identified from the current study

**Fig 2:**
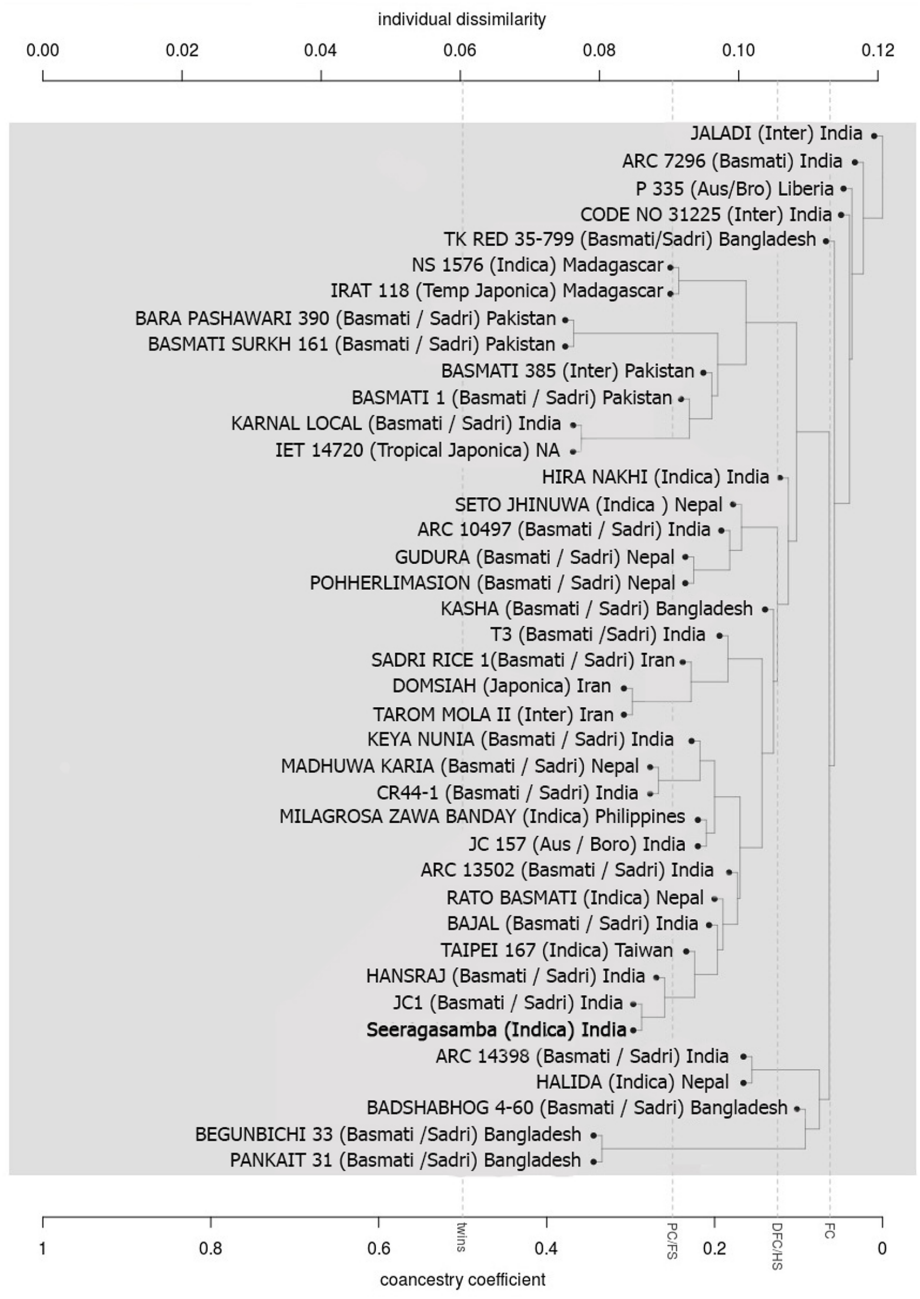
The dendrogram showing the relationship between Seeragasamba and 39 aromatic rice varieties based on whole genome SNPs. The SNP data for the 39 aromatic rice varieties were extracted from the Rice SNP-Seek Database. Name of the aromatic rice variety followed by its type in bracket, and its geographical origin are given for each entry.

The new allele identified here, *badh2-p*, was present in 13 varieties (approximately 17%). Interestingly, 17 varieties with the *badh2-E7* allele also had *badh2-p* allele bringing the total number varieties with *badh2-p* allele to 30 (approximately 40%). This is higher than the frequency of *badh2-E7* allele in aromatic rice varieties. The presence of the *badh2-p* allele is not significant in the varieties with the *badh2-E7* allele, which represents a loss-of-function mutation. Among the varieties that did not have any apparent loss-of-function mutation (InDels and non-sense SNPs in coding regions), the frequency of *badh2-p* allele was approximately 30% (13 out of 50 varieties). The varieties with *badh2-p* allele included both indica and japonica types. Similarity analysis of 40 aromatic rice varieties using genome-wide SNPs, showed grouping of Seeragasamba with six other aromatic rice varieties from India, Nepal and Taiwan, all of which contained the 8 bp insertions in the promoter region (Fig 2 and Table 2). Previous studies might have underestimated the frequency of the *badh2-p* allele in aromatic rice varieties because of targeted screening for *badh2-E7* and other alleles rather than sequencing the whole gene or genome. This study strongly indicates that *badh2-p* should be included in the screening for fragrance alleles in aromatic rice varieties. Indels near the *cis* elements can greatly influence the expression of the cognate genes. Genes with differential expression harbor more indels in the promoter, especially between 500–1500 bp upstream regions, than those that are not differentially expressed [34]. However, experimental promoter analysis is needed to establish effect of *badh2-p* allele on the expression of BADH2 gene.
